# Dicer-mediated miRNA processing is not involved in controlling muscle mass during muscle atrophy

**DOI:** 10.1038/s41598-021-98545-0

**Published:** 2021-09-29

**Authors:** Satoshi Oikawa, Jaehoon Shin, Takao Akama, Takayuki Akimoto

**Affiliations:** 1 Faculty of Sport Sciences, Waseda University, Saitama, 359-1192 Japan; 2 Graduate School of Sport Sciences, Waseda University, Saitama, 359-1192 Japan

**Keywords:** Cell biology, Molecular biology

## Abstract

Muscle atrophy occurs in a variety of physiological and pathological conditions. Specific molecular networks that govern protein synthesis and degradation play important roles in controlling muscle mass under diverse catabolic states. MicroRNAs (miRNAs) were previously found to be regulators of protein synthesis and degradation, and their expressions in skeletal muscle were altered in muscle wasting conditions. However, functional roles of miRNAs in muscle atrophy are poorly understood. In this study, we generated tamoxifen-inducible *Dicer* knockout (iDicer KO) mice and subjected them to 2 weeks of single hindlimb denervation. The expression of *Dicer* mRNA was significantly reduced in muscle of the iDicer KO mice compared to that of WT mice. The loss of *Dicer* moderately reduced levels of muscle-enriched miRNAs, miR-1, miR-133a and miR-206 in both innervated and denervated muscles of the iDicer KO mice. We also found that the extent of denervation-induced muscle atrophy as well as changes of signaling molecules related to protein synthesis/degradation pathways in the iDicer KO mice were comparable to these in WT mice. Taken together, *Dicer* knockout in adult skeletal muscle did not affect denervation-induced muscle atrophy.

## Introduction

Muscle atrophy occurs in diverse physiological and pathological conditions, including aging (sarcopenia), cancer-associated cachexia, diabetes and neurodegenerative diseases. The loss of muscle mass and strength is a major cause of frailty and mortality in elderly people and patients with muscular diseases such as sarcopenia and amyotrophic lateral sclerosis (ALS). Thus, understanding the molecular mechanisms that maintain muscle mass would provide novel therapeutic targets for treatment of muscle atrophy.

It is well recognized that muscle atrophy is induced by an imbalance between protein synthesis and degradation^1,2^. The Akt/mTOR signaling pathway, which is a potent activator of protein synthesis, was attenuated during muscle atrophy, whereas genetic hyper-activation of this pathway induced muscle hypertrophy and prevented denervation-induced muscle atrophy^3^. Comprehensive expression profiling identified a group of genes that were up-regulated in atrophying muscles, which are called “atrogenes”^4^. Accelerated protein breakdown by muscle-specific E3 ubiquitin ligases, muscle-specific RING-finger 1 (MuRF1)^5^ and atrogin-1 (also known as MAFbx)^6^, leads to skeletal muscle loss in various atrophic conditions. Indeed, mutant mice disrupted either MuRF1 or atrogin-1 showed resistance to muscle atrophy caused by denervation^5^. Autophagy is a fundamental process that maintains protein and organelle homeostasis through their degradation in lysosome^7^, and its dysfunction exacerbated muscle atrophy with accumulation of abnormal mitochondria and ubiquitinated-proteins during denervation and fasting^8^. These findings indicated that specific signaling pathways play important roles in controlling muscle mass under catabolic conditions, while the molecular mechanisms underlying muscle atrophy have not been fully established.

MicroRNAs (miRNAs) are ~ 22 nucleotides non-coding RNAs that post-transcriptionally inhibit their target messenger RNA (mRNA) expressions^9^. It has been established that miRNAs act as a fundamental component in different biological processes such as development and systemic diseases, across species^10^. Biogenesis of miRNAs is accomplished by multiple steps in the nucleus and the cytoplasm^11^. In the cytoplasm, endoribonuclease III Dicer recognizes precursors of miRNAs (pre-miRNAs), which are exported from the nucleus, and processes them into double-stranded miRNAs. The Dicer-mediated processing of pre-miRNAs enables them to be loaded onto Argonaute proteins (Ago), resulting in assembly of an RNA–protein complex, called RNA-induced silencing complex (RISC). RISC complex targets miRNA-binding sites in the 3′-UTR of mRNAs to induce mRNAs decay and/or their translational repression. It has also well known that the Dicer-mediated miRNAs processing is a vital step in regulating mature miRNAs expression, which is indispensable for development and tissue homeostasis in vertebrates^12–18^.

Previous studies have demonstrated that several miRNAs involved in controlling muscle mass and miRNA expression profiling identified that a subset of miRNAs expressed aberrantly in skeletal muscle under wasting conditions^19–21^. miR-455-3p was decreased in skeletal muscle of aged (24-month-old) mice compared to young (6-month-old) mice^21^. Expression of miR-455-3p induced hypertrophy in C2C12 myotubes^21^ and directly repressed expression of phosphatase and tensin homolog (PTEN), an inhibitor of protein synthesis pathway^22^. miR-486 was up-regulated during muscle atrophy and inhibited expressions of PTEN and FoxO1a^23^. miR-206 was strongly up-regulated in ALS patients and G93A-SOD1 transgenic mice, which recapitulate the phenotypes of human ALS^20,24^. We also found that miR-23 targeted MuRF1 and atrogin-1, and overexpression of miR-23 attenuated dexamethasone-induced muscle atrophy via translational inhibition of these two ubiquitin ligases in myotubes^25^. However, the functional relevance of miRNAs in muscle atrophy in vivo are poorly understood. Therefore, we generated mutant mice in which *Dicer* is genetically disrupted in a tamoxifen-dependent manner to deplete mature miRNAs thoroughly and subjected them to 2 weeks of denervation by transection of sciatic nerve to induce hindlimb muscle atrophy in the present study.

## Results

### Body weight, muscle weight and muscle strength in tamoxifen-inducible *Dicer* knockout mice

To examine the role of miRNAs in controlling muscle mass and strength in adult mice, we used tamoxifen-inducible *Dicer* knockout (iDicer KO) mice^26,27^. We administered tamoxifen to WT and the iDicer KO mice to delete *Dicer* gene and measured their body weight, muscle weight and maximum muscle strength under sedentary condition. We found that the iDicer KO mice showed significantly lower body weight than WT mice at 4 weeks after tamoxifen injection (Fig. 1A). No differences in the weight of tibialis anterior (TA), extensor digitorum longus (EDL), gastrocnemius (Gas), plantaris and soleus muscles were observed between WT and iDicer KO mice 2 and 4 weeks after injection (Fig. 1B,C). Maximum grip strength at 4 weeks after tamoxifen-induced recombination was also comparable in both genotypes (Fig. 1D).

**﻿Figure 1:**
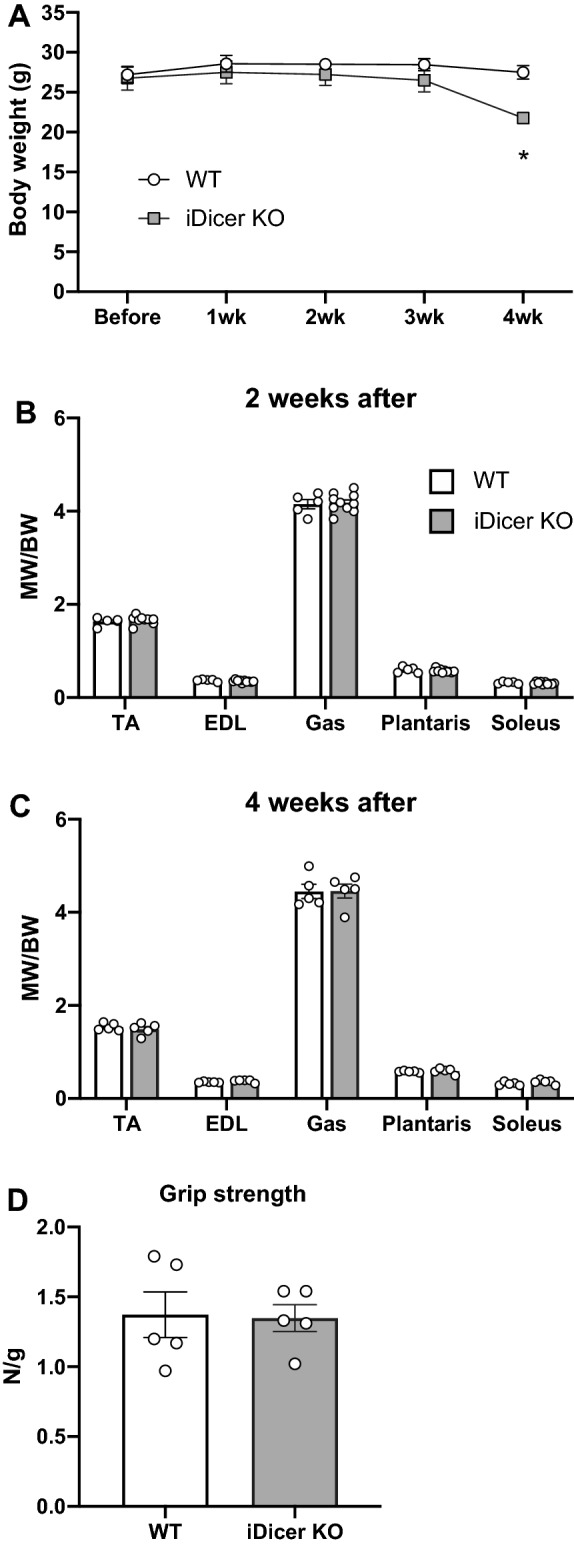
Body weight, skeletal muscle weight and muscle strength in tamoxifen-inducible *Dicer* knockout mice. (**A**) At 4 weeks post-tamoxifen injection, there were a significant difference in body weight between WT and iDicer KO mice (n = 4–5). (**B**,**C**) At 2 and 4 weeks after tamoxifen injection, no differences in skeletal muscle weight were observed in iDicer KO mice in comparison with WT mice (n = 4–5). (**D**) Grip strength at 4 weeks post-tamoxifen treatment also did not show significant differences between WT and iDicer KO mice (n = 5). *P < 0.05.

### The expression of *Dicer* and muscle-enriched miRNAs in muscle of the iDicer KO mice after 2 weeks of denervation

**Figure 2:**
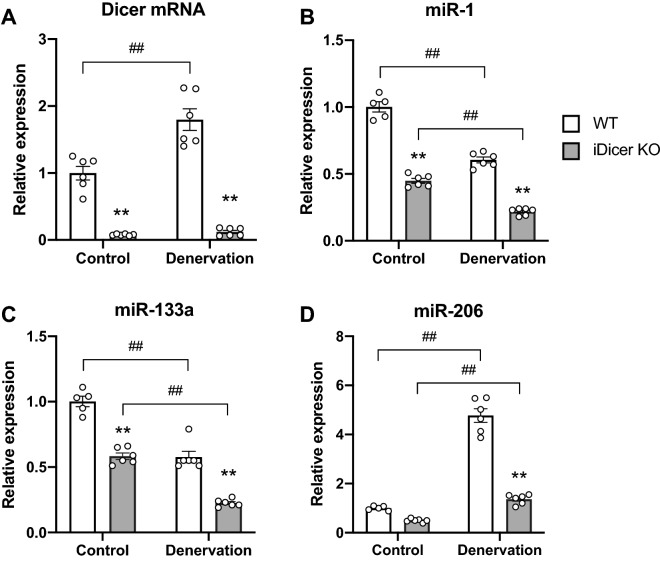
*Dicer* mRNA and muscle-specific miRNAs levels in plantaris muscles after 2 weeks of denervation. (**A**) A significant reduction in *Dicer* mRNA levels was observed in both control and denervated-plantaris muscles of iDicer KO mice. Two weeks of denervation increased *Dicer* mRNA levels in the plantaris muscles from WT mice (n = 6). (**B**–**D**) Tamoxifen-inducible deletion of *Dicer* significantly reduced the expression of skeletal muscle-enriched miRNAs, miR-1, miR-133a and miR-206 in both innervated and denervated-plantaris muscles in the iDicer KO mice (n = 5–6). **P < 0.01 vs WT, ^##^P < 0.01.

**Figure 3:**
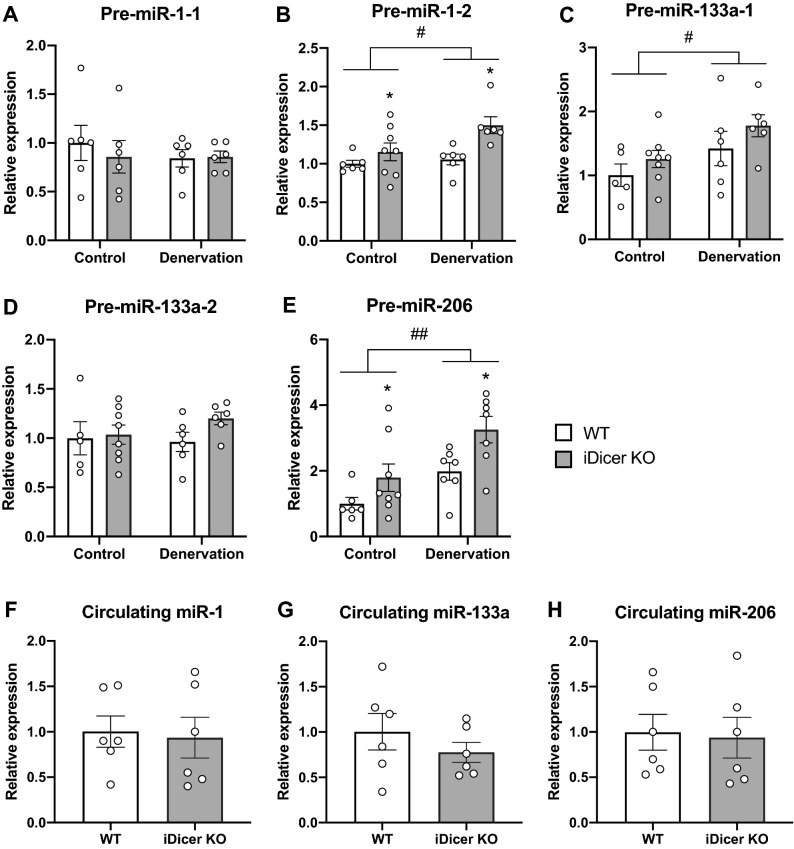
The expression levels of pre-miRNAs in plantaris muscles and circulating miRNAs in plasma after 2 weeks of denervation. (**A**–**E**) Expression of pre-miR-1-1, pre-miR-1-2, pre-miR-133a-1, pre-miR-133a-2 and pre-miR-206 (n = 4–8) after 2 weeks of denervation. Two weeks of denervation increased pre-miR-1-2, miR-133a-1 and pre-miR206 levels in the plantaris muscles in both genotypes. In both innervated and denervated Dicer knockout muscles, increased pre-miR1-2 and pre-miR-206 levels were observed. (**F**–**H**) Tamoxifen-inducible deletion of *Dicer* did not affect circulating miR-1, miR-133a and miR-206 levels in plasma (n = 6). *P < 0.05 vs WT, ^#^P < 0.05, ^##^P < 0.01.

To determine the role of miRNAs in muscle wasting, WT and iDicer KO mice were subjected to denervation-induced muscle atrophy 1 week after tamoxifen treatment. We first examined the expression of *Dicer* mRNA and muscle-specific miRNAs in plantaris muscle of WT and iDicer KO mice after denervation. Consistent with a previous work from our group^26^, the expression of *Dicer* mRNA was significantly reduced in both control and denervated plantaris muscles of iDicer KO mice in comparison to those of WT mice (Fig. 2A). Also, loss of *Dicer* mildly but significantly reduced the levels of miR-1 and miR-133a, that are specifically and highly expressed in striated muscles, in both control and denervated muscles of iDicer KO mice (Fig. 2B,C). Two weeks of denervation increased the *Dicer* mRNA levels in the plantaris muscle from WT mice (Fig. 2A) and reduced miR-1 and miR-133a levels in both WT and iDicer KO muscles (Fig. 2B,C). Denervation also increased level of miR-206 expression in both WT and iDicer KO mice (Fig. 2D). However, the miR-206 expression in the denervated plantaris muscle of iDicer KO mice was significantly lower than that of WT mice (Fig. 2D). In addition, we measured precursors of these miRNAs (pre-miRNAs) after denervation and observed an increase of pre-miRNAs including pre-miR-1-2 and pre-miR-206 in the iDicer KO mice (Fig. 3A–E). We also quantified circulating miRNAs levels, and the expression levels of circulating miR-1, miR-133a and miR-206 in plasma were comparable in WT and the iDicer KO mice (Fig. 3F–H).

### Denervation-induced atrophy of slow-twitch soleus and fast-twitch plantaris muscles in the iDicer KO mice

**Figure 4:**
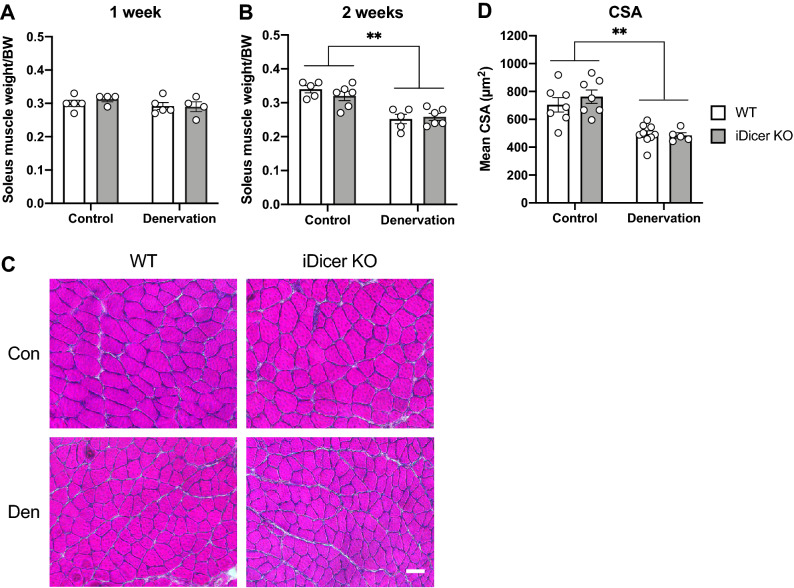
Denervation-induced atrophy of slow-twitch soleus muscles in the iDicer knockout mice. (**A**,**B**) Changes of soleus muscle weight after 1 week (**A**) and 2 weeks (**B**) of denervation. At 2 weeks after denervation, soleus muscle weight was significantly reduced in both WT and iDicer KO mice (Denervation, P < 0.01), while inducible *Dicer* deletion did not affect denervation-induced soleus muscle atrophy (n = 4–6). (**C**) Representative images of sections of control and denervated soleus muscles stained with hematoxylin–eosin (H&E). (**D**) Denervation caused a reduction in cross-sectional areas (CSA) of soleus muscle fibers in both WT and iDicer KO mice (Denervation, P < 0.01). There were no differences in muscle fiber atrophy between genotypes (n = 5–10). **P < 0.01. Scale bar = 50 µm.

**Figure 5:**
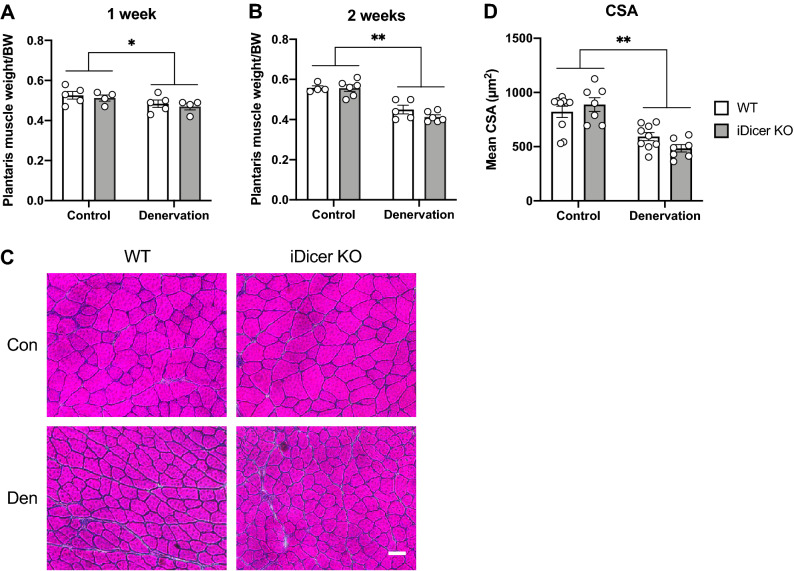
Denervation-induced atrophy of fast-twitch plantaris muscles in the iDicer knockout mice. (**A**,**B**) Changes of plantaris muscle weight after 1 week (**A**) and 2 weeks (**B**) of denervation. The plantaris muscle weight was significantly decreased in both genotypes at 1 week (Denervation, P < 0.05) and 2 weeks after denervation (Denervation, P < 0.01). Deletion of *Dicer* in adult muscles did not also affect denervation-induced plantaris muscle atrophy (n = 4–6). (**C**) Representative images of H&E staining in control and denervated plantaris muscles of WT and iDicer KO mice. (**D**) Mean fiber CSAs of denervated plantaris muscles did not alter between WT and iDicer KO mice (Denervation, P < 0.01) (n = 7–10). *P < 0.05, **P < 0.01. Scale bar = 50 µm.

We next determined if the reduction in expressions of *Dicer* and miRNAs affects denervation-induced muscle atrophy. At 2 weeks after denervation, soleus muscle weight was significantly reduced in both WT and iDicer KO mice, whereas no significant differences were seen between the genotypes (Fig. 4A,B). Histological analysis also showed the muscle fiber cross-sectional area (CSA) of denervated muscles was similar between WT and iDicer KO mice (Fig. 4C,D). We also found that the plantaris muscle weight as well as CSA were significantly reduced in both genotypes at 1 week and 2 weeks after denervation, while there were no significant differences between the two groups (Fig. 5A–D).

### Protein expressions in relation to protein synthesis and degradation pathways in the iDicer KO mice following denervation

**Figure 6:**
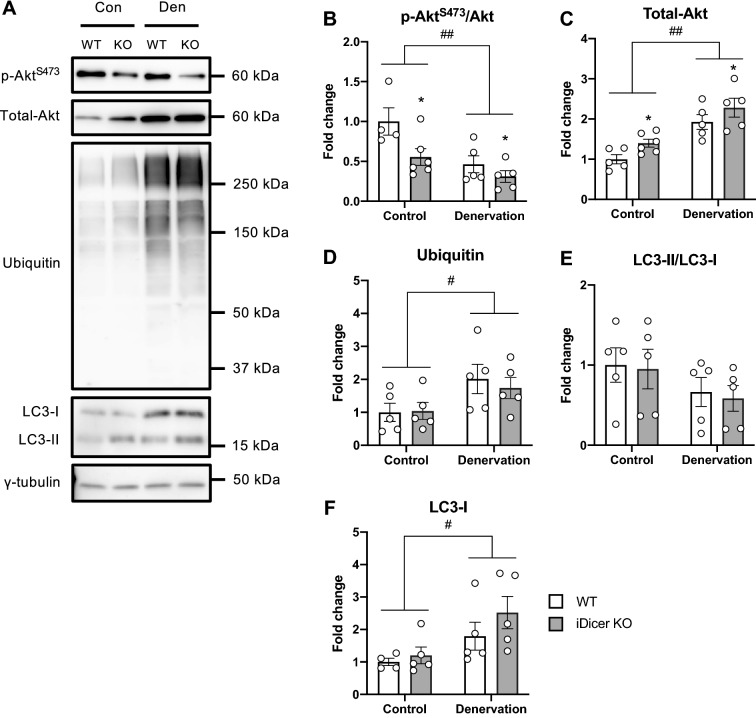
Protein expressions related to protein synthesis and degradation pathway in the iDicer knockout mice following denervation. (**A**) Representative blotting images for phosphorylated-Akt^S473^, total-Akt, Ubiquitinated-proteins, LC3-I/II and γ-tubulin (n = 4–6). (**B**) The ratio of the phosphorylated-Akt^S473^/total-Akt in the plantaris muscle from iDicer KO mice were lower than those from WT mice (Genotype, P < 0.05). Their levels in the plantaris muscles of WT and iDicer KO mice were decreased after 2 weeks of denervation (Denervation, P < 0.01). (**C**) Hindlimb denervation increased the levels of total-Akt proteins in both WT and iDicer KO mice (Denervation, P < 0.01), and the levels in the iDicer KO muscles were higher than in WT muscles (Genotype, P < 0.05). (**D**) There was a significant increase in the ubiquitinated-proteins levels in both WT and iDicer KO mice after denervation (Denervation, P < 0.05), whereas the differences were not seen between genotypes. (**E**) No significant differences were observed in the LC3-II/LC3-I ratio between WT and iDicer KO mice after 2 weeks of denervation. (**F**) The upregulation of LC3-I following denervation was comparable in both WT and iDicer KO mice (Denervation, P < 0.05). *P < 0.05 vs WT, ^#^P < 0.05, ^##^P < 0.01.

We assessed protein expressions associated with protein synthesis and degradation pathways in WT and iDicer KO mice after 2 weeks of denervation (Fig. 6A). The ratio of phosphorylated-Akt^S473^/total-Akt in plantaris muscle from iDicer KO mice were lower than that from WT mice and their levels decreased in both WT and iDicer KO mice following denervation (Fig. 6B). Hindlimb denervation increased levels of total-Akt protein in both WT and iDicer KO mice, and their levels were increased in both innervated and denervated plantaris muscles of iDicer KO mice (Fig. 6C). We observed that ubiquitinated protein levels were significantly increased in denervated WT and *Dicer* knockout muscles but there was no difference between genotypes (Fig. 6D). The ratio of LC3-II/LC3-I, which is a marker of autophagic flux, did not change between genotypes (Fig. 6E), while the LC3-I levels were increased in both groups after 2 weeks of denervation (Fig. 6F).

## Discussion

The finding that miRNAs were differentially expressed in skeletal muscle under catabolic conditions and modulated protein synthesis/degradation signaling pathways, promoted us to determine roles of miRNAs in regulating muscle mass during muscle atrophy. However, the decreased miRNAs did not affect muscle atrophy upon denervation in the present study (Figs. 4 and 5). It was also reported recently that muscle-specific *Dicer* knockout mice did not show clear difference during hindlimb suspension-induced muscle atrophy^28^. Collectively, these data denoted that muscle atrophy would not be affected by the modest, global decrease in miRNAs following *Dicer* inactivation in adult skeletal muscle.

On the contrary, several studies have shown that the manipulation of a specific miRNA in skeletal muscle affected muscle mass and atrophy. miR-29b was induced by various models of muscle wasting and promoted muscle atrophy by inhibiting IGF-1 and PI3K mRNA expressions^29^. Moreover, overexpression of miR-206 caused a reduction in fiber size in both innervated and denervated muscles, whereas knockdown of miR-206 induced hypertrophy in innervated muscle^19^. In the present study, we observed no change in muscle mass and morphology of the iDicer KO mice upon denervation (Figs. 4 and 5), although miR-206 in denervated muscle of the iDicer KO mice was approximately 70% lower than that in WT muscle (Fig. 2D). Taken together, this mouse model may not be suitable for determining the exact roles of miRNAs in adult skeletal muscle. It is conceivable that future experiments utilizing mutant mice in which specific miRNAs are disrupted in skeletal muscles are able to provide precise functions of miRNAs in muscle wasting.

Interestingly, our data showed that genetic deletion of *Dicer* significantly repressed an increase of miR-206 with accumulation of pre-miR-206 in response to denervation (Figs. 2D and 3E), indicating that the Dicer-dependent miRNAs biogenesis was clearly inhibited in skeletal muscle. Although it is generally recognized that the processing of pre-miRNAs by Dicer is essential for mature miRNA expressions, several studies have shown that some miRNAs were able to be produced in a Dicer-independent manner^30–33^. The maturation of miR-451, which plays an important role in erythropoiesis, requires direct processing by Ago2 rather than that by Dicer^30–32^. Similarly, a previous study reported that pre-miRNAs were directly loaded onto Ago2 proteins, allowing maturation and expression of 5p-miRNAs through unidentified mechanisms in *Dicer* knockout cells^33^. Therefore, the Dicer-independent miRNAs synthesis is likely to contribute to mature miRNA expressions in the iDicer KO mice.

We found that tamoxifen-inducible knockout of *Dicer* in mice showed a reduction in their body weight at 4 weeks after tamoxifen injection (Fig. 1A) and led to death (data not shown), indicating Dicer and miRNAs are essential for maintaining physical function in adult mice. It has also become clear that Dicer and miRNAs have critical roles in controlling muscle development, disease and regeneration^20,34–36^. Genetic inactivation of *Dicer* specifically in muscle during embryogenesis resulted in skeletal muscle hypoplasia and perinatal death^34^. A previous work from Rando’s group demonstrated that muscle satellite cells (SCs)-specific, tamoxifen-inducible deletion of *Dicer* severely impaired SCs function and muscle regeneration^35^. By contrast, Vechetti et al. reported that tamoxifen-inducible, HSA-Cre^ERT^-mediated skeletal muscle-specific *Dicer* knockout mice showed no differences in muscle mass and morphology^28^, which is consistent with our results. Intriguingly, in this genetic model, the expressions of miR-1, miR-133a and miR-206 were only decreased by ~ 30–40%, although *Dicer* mRNA was significantly reduced in skeletal muscle following tamoxifen treatment^28^. Taken together, these data indicate that the remaining miRNAs in the *Dicer*-deficient skeletal muscles might be sufficient to maintain muscle mass and function.

The levels of miR-1, miR-133a and miR-206 in innervated iDicer KO muscle reduced by almost 50% at 3 weeks after tamoxifen injection (Fig. 2B–D), suggesting half-life of these miRNAs in adult skeletal muscles were approximately > 21 days. A previous study reported that miR-208 also showed longer half-life in vivo (> 12 days)^37^ compared to that of mRNAs. Recently, it has been shown that miRNAs had greater stability than canonical mRNAs in culture cells^38–40^. Metabolic labeling of nascent RNAs with a uridine analog and high-throughput RNA-sequencing analysis demonstrated that their median half-life was about ten-fold greater than that of mRNAs^38^. However, the dynamics of miRNAs turnover and its half-life in vivo has not yet defined. Likewise, although the molecular basis of miRNAs biogenesis pathway has been established, little is known about the mechanisms that control the stability and degradation of miRNAs. In the future studies, it will be needed to determine half-life of miRNAs in skeletal muscle in vivo and the mechanisms regulating their stability and decay.

Circulating miRNAs might be another concern to be involved in expression of miRNAs in skeletal muscle of the iDicer KO mice. Adipose tissue was found to be a major source of exosomal miRNAs in the circulation, and fat-derived exosomal miRNA, miR-99b, regulated *Fgf21* mRNA expression in the liver^41^. Moreover, exosomal miR-27a released from adipose tissue in obese mice accumulated in skeletal muscle and impaired insulin signaling by regulating the expression of peroxisome proliferator-activated receptor γ (*PPARγ*) gene^42^. These results suggest that skeletal muscle has the ability to take up exosomal miRNAs from the circulation. miR-1 and miR-133a that are expressed specifically in cardiac and skeletal muscles^43^, were detected in blood of patients with cardiovascular disease and healthy humans^44,45^. Since we could not observe the significant reduction of circulating miR-1, miR-133a and miR-206 expression levels in plasma in the iDicer KO mice (Fig. 3F–H), these circulating miRNAs might be expressed in skeletal muscle of the iDicer KO mice.

Nie et al. demonstrated that miR-133a-null mice displayed hyperactivation of protein synthesis pathway with increased levels of phosphorylated Akt and S6 kinase^46^. Moreover, genetic deletion of miR-378, a muscle-enriched miRNA, provoked muscle atrophy with impaired autophagy by targeting phosphoinositide-dependent protein kinase 1 (PDK1)^47^. In this study, we observed that the ratio of phosphorylated-Akt/total-Akt was decreased in denervated muscles with no differences in these levels among the genotypes (Fig. 6B), probably owing to increased total-Akt protein content in denervated muscles (Fig. 6C). In addition, no difference in the poly-ubiquitinated proteins levels (Fig. 6D) and the LC3-II/I ratio (Fig. 6E) between WT and the iDicer KO muscles was seen in response to denervation. These results support the data showing that the extent of muscle atrophy in the iDicer KO mice was comparable to that of WT mice (Figs. 4, 5). Collectively, although miRNAs must be an important mediator for protein synthesis and degradation, these pathways could not be disturbed by a moderate reduction in miRNAs in the iDicer KO mice during muscle atrophy.

## Materials and methods

### Animal experiments

All mice were housed in temperature-controlled quarters (22 °C) under 12-h light–dark cycles with ad-libitum access to water and food. Tamoxifen-inducible *Dicer* knockout (iDicer KO) mice were obtained by intercrossing *Dicer*-floxed mice^34^ with CAG-Cre^ERT2^ mice^48^ and maintained on a C57/BL6 background, as previously described^26,27^. At the age of 10 weeks, female mice were intraperitoneally treated with tamoxifen (1 mg/day) (Sigma-Aldrich, St Louis, MO) for 5 consecutive days to induce Cre-mediated recombination. Tamoxifen-treated Cre-negative littermates (*Dicer*-floxed mice) were used as controls (WT). Maximum grip strength of all limbs was measured using the digital force gauge (DST-50 N; Imada, Aichi, Japan) as previously described^26,49^. Denervation was carried out by cutting a 3-mm length of the sciatic nerve of the left limb under isoflurane (2%) anesthesia and the right limb was used as the control^50^. Soleus and plantaris muscles were harvested 7 or 14 days after sciatic nerve transection, frozen in liquid nitrogen for following experiments. The procedures of animal experiments were approved by the Animal Care and Use Committees of Waseda University, Japan (numbers: 2017-A103a, 2018-A122, 2019-A108, 2020-A012) and performed in accordance with the institutional and national guidelines. This study was carried out in compliance with the ARRIVE guidelines.

### Blood sampling and plasma RNA isolation

After 2 weeks denervation, blood samples were obtained from the inferior vena cava and plasma samples were prepared by centrifugation and stored them at − 80 °C until use. Total RNA was isolated from plasma using miRNeasy Mini Kit (Qiagen, Valencia, CA, USA) according to the manufacturer’s instructions, as previously described^51^. Briefly, 200 µl of the plasma sample was mixed with five volumes of QIAzol reagent (Qiagen, Valencia, CA, USA). Synthetic *C. elegans* miRNAs, cel-miR-39, cel-miR-54 and cel-miR-238 (synthetic RNA oligonucleotides, Fasmac, Odawara, Japan), were added to each sample (25 nM of each oligonucleotide in a 5 µl total volume) to normalize the sample-to-sample variation in RNA isolation. Then, chloroform was added to the mixture of plasma, QIAzol and synthetic *C. elegans* miRNAs, cel-miR-39, cel-miR-54 and cel-miR238, in equal volume to the plasma and vortexed well. After centrifugation for 15 min at 12,000×*g* at 4 °C, the upper aqueous phase was transferred to a new collection tube, mixed with 1.5 volumes of 100% ethanol, and transferred to an RNeasy Mini spin column. After centrifugation for 1 min at 15,000×*g* at 25 °C, 700 µl of Buffer RWT and 500 µl of Buffer RPE were added to the RNeasy Mini spin column followed by centrifugation for 1 min at 15,000×*g* at 25 °C to wash the column. Lastly, total RNA was eluted with 50 µl of RNase-free water and stored at − 80 °C.

### Real-time PCR analysis

Total RNA was extracted from plantaris muscle using ISOGEN II (Nippon gene, Tokyo, Japan). ReverTra Ace qPCR RT Kit (Toyobo, Osaka, Japan) and Thunderbird SYBR qPCR Mix (Toyobo) were used for quantification of mRNA expression. The following primers were used for *Dicer*: forward, 5′-CACACGCCTCCTACCACTACAACAC-3′; reverse, 5′-CCGTGGGTCTTCATAAAGGT-3′ and *GAPDH*: forward, 5′-AAATGGTGAAGGTCGGTGTG-3′; reverse, 5′-TGAAGGGGTCGTTGATGG-3′.

*GAPDH* was used as an internal control. The TaqMan MicroRNA Reverse Transcription Kit (Applied Biosystems, Foster City, CA), and TaqMan MicroRNA Assays (Applied Biosystems) were used for real-time PCR quantification of mature miRNA expression. Quantitative real-time PCR analysis for mRNA and miRNAs was conducted using the Applied Biosystems StepOnePlus Real-Time PCR system (Applied Biosystems) at 95 °C for 10 min, followed by 40 cycles of 95 °C for 15 s and 60 °C for 1 min^26^. The small RNA *U6* and *cel-miR-39* were used as internal standards to normalize the miRNA expression for plantaris muscles and plasma samples, respectively. Catalog numbers of TaqMan MicroRNA assays were as follows: miR-1 (no. 002222), miR-133a (no. 002246), miR-206 (no. 000510) and cel-miR-39 (no. 000200).

### Semi-quantitative RT-PCR

To quantify the expression of precursor-miRNAs (pre-miRNAs), SuperScript III reverse transcriptase (Thermo Fisher Scientific, Colorado, USA) and Ex Taq HS (Takara, Osaka, Japan) were used for semi-quantitative RT-PCR analysis. Briefly, the mixture containing 500 ng of total RNA, 1 µl of 40 pM miRNA-specific reverse primer, 1 µl of 10 mM dNTP mix and nuclease-free water (up to 12 µl) was heated at 65 °C for 5 min and placed it on ice for at least 1 min. Then, 4 µl of 5 × First-Strand Buffer, 1 µl of 0.1 M DTT, 1 µl of RNase inhibitor and 1 µl of SuerScript III Reverse Transcriptase were added to the mixture. The reaction mixture was incubated at 55 °C for 60 min and heated at 70 °C for 15 min. The cDNA was amplified by standard PCR protocol using Ex Taq HS. The PCR products were electrophoresed on 2% agarose gels containing ethidium bromide, and images were acquired by the LAS-3000 imaging system (GE healthcare, Little Chalfont, UK) and analyzed by image j software (National Institute of Health, Bethesda, MD). The following primers were used for *pre-miR-1-1*: forward, 5′-GCTTGGGACACATACTTCTTT-3′; reverse, 5′-TTCCATAGCTTAGCAGGTTCA-3′, *pre-miR-1-2*: forward, 5′-AGCACATACTTCTTTATGTACCC-3′; reverse, 5′-TTCTTTACATTCCATAGCACTGAA-3′, *pre-miR-133a-1*: forward, 5′-AAAGCTGGTAAAATGGAACCAA-3′; reverse, 5′-TGCATAGCTACAGCTGGTTGA-3′, *pre-miR-133a-2*: forward, 5′-AATGCTTTGCTGAAGCTGGT-3′; reverse, 5′-CTGGTTGAAGGGGACCAAAT-3′, *pre-miR-206*: forward, 5′-CCAGGCCACATGCTTCTTTA-3′; reverse, 5′-CCACACACTTCCTTACATTCCA-3′ and *GAPDH*: forward, 5′-GACCCCTTCATTGACCTCAAC-3′; reverse, 5′-TAAGCAGTTGGTGGTGCAGGA-3′. *GAPDH* was used as an internal standard.

### Histology and fiber size measurements

Cryosections (10 µm) of soleus and plantaris muscles embedded in OCT-compound were stained with hematoxylin (Sigma-Aldrich) and eosin (Wako Chemicals, Osaka, Japan). Briefly, muscle sections were incubated with hematoxylin solution for 30 min to stain the nuclei and blued in warm tap water for 10 min and dehydrated with 90% ethanol. The sections were then incubated with eosin solution for 15 s to stain the cytoplasm and immersed in 90% ethanol for 20 s, 100% ethanol for 20 s, and mounted on a glass slide with the mounting medium. Images of the H&E-stained cross-sections were taken with an IX-70 microscope (Olympus, Tokyo, Japan) equipped with a DS-Ri1 digital camera (Nikon, Tokyo, Japan). The cross-sectional areas (CSA) of muscle fibers were measured using ImageJ software (National Institute of Health, https://imagej.nih.gov/ij/).

### Western blot analysis

Western blot analysis was performed as described elsewhere^26^. Briefly, plantaris muscles were lysed in complete protein loading buffer containing 50 mM Tris–HCl (pH 6.8), 1% SDS, 10% glycerol, 20 mM dithiothreitol (DTT), 127 mM 2-mercaptoethanol, and 0.01% bromophenol blue, supplemented with protease inhibitors (Roche, Basel, Switzerland) and phosphatase inhibitors (Sigma-Aldrich). The protein contents of plantaris muscle homogenates were measured with an *RC DC* Protein Assay Kit (Bio-Rad, Hercules, CA) according to the manufacture’s instruction. Total proteins (40–60 µg) were electrophoresed on SDS-PAGE gels (10 and 12%) and transferred to a nitrocellulose membrane, and the signals were immunodetected with Amersham ECL Prime Western Blotting Detection Reagent (Cytiva, Marlborough, MA) using the LAS-3000 Imaging System (Fuji Film, Tokyo, Japan). The following antibodies were used: Akt (#9272, 1:1000, Cell Signaling Technology (CST), Danvers, MA), phospho-Akt^Ser473^ (#4060, 1:1000, CST), LC3-I/II (#4108, 1:1000, CST), ubiquitin (sc-8017, 1:1000, Santa Cruz Biotechnology, Dallas, TX) and γ-tubulin (T3320, 1:1000, Sigma-Aldrich). Anti-Rabbit IgG, HRP-Linked F (ab′)_2_ Fragment (NA9340, Cytiva) and anti-mouse IgG-κ-binding protein-HRP (sc-516102, Santa Cruz Biotechnology) were used as secondary antibodies. γ-tubulin was used as an internal control. The protein levels were quantified by densitometry using ImageJ software (National Institute of Health, https://imagej.nih.gov/ij/) (Figure S1).

### Statistical analysis

Data were presented as means ± SE. Statistical significance (P < 0.05) was determined with Student’s *t* test for comparisons of two groups or with two-way ANOVA followed by the Tukey’s multiple comparisons test for multiple comparisons (genotype × denervation). All statistical analyses were carried out using GraphPad Prism 8 (Version 8.4.3, GraphPad Software, La Jolla, CA, https://www.graphpad.com/scientific-software/prism/).

## Supplementary Information

Supplementary Information.
