# Supplementary material for: Dicer-mediated miRNA processing is not involved in controlling muscle mass during muscle atrophy
Source: Sci Rep. 2021 Sep 29;11:19361. doi: 10.1038/s41598-021-98545-0 (PMC8481297; doi:10.1038/s41598-021-98545-0)
Supplement: Supplementary file 1 — Supplementary Information. [file 41598_2021_98545_MOESM1_ESM.pdf]

Supplementary Figure S1

Representative blot images displayed in Fig. 6A

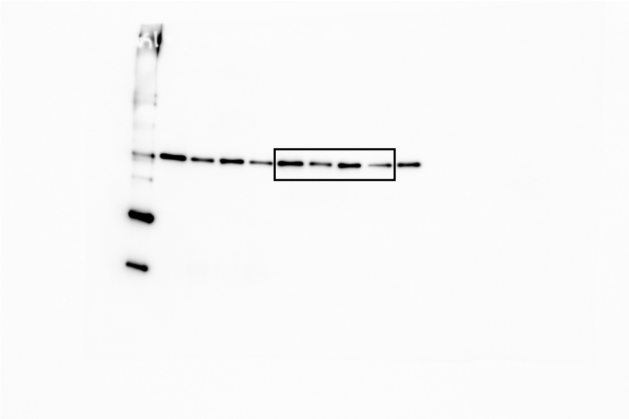

p-Akt^{S473}

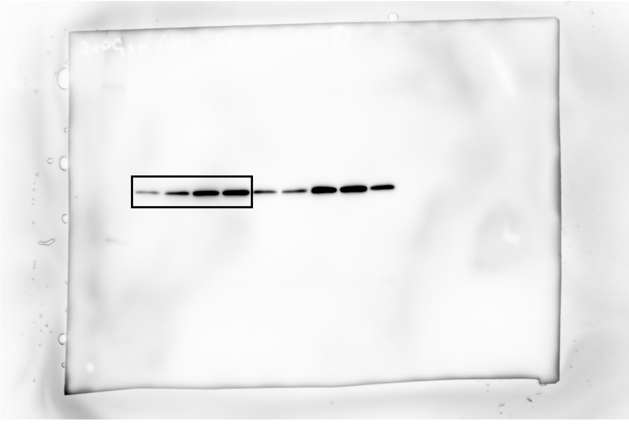

total-Akt

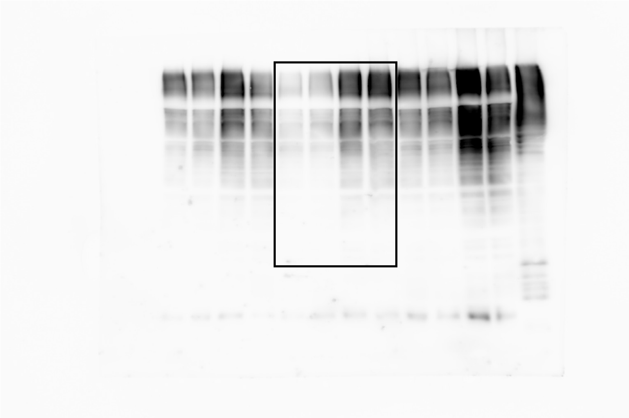

Ubiquitin

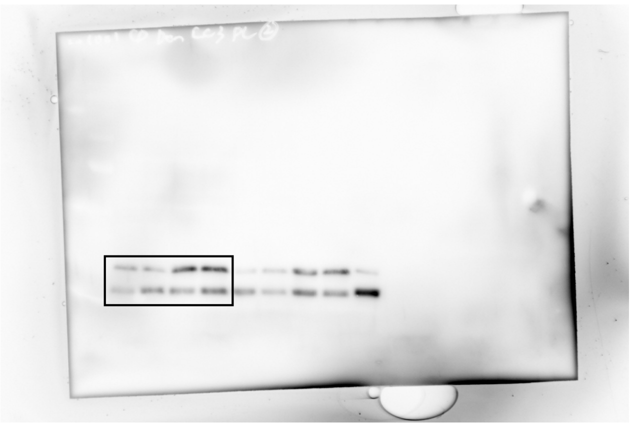

LC3-I/II

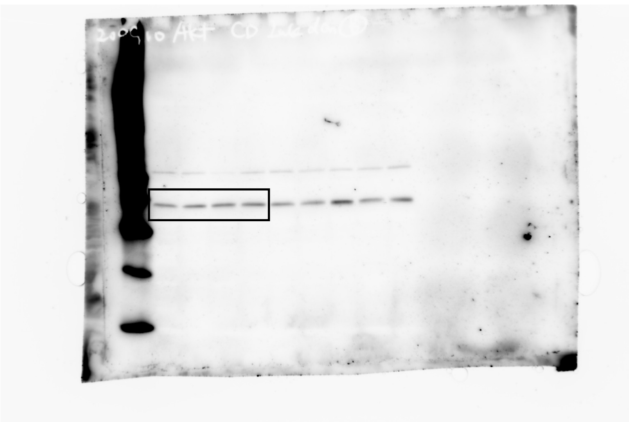

γ-tubulin
